# Laser Treatment for Diabetic Macular Edema in the 21^st^ Century

**DOI:** 10.2174/1573399810666140402123026

**Published:** 2014-03

**Authors:** Pedro Romero-Aroca, Javier Reyes-Torres, Marc Baget-Bernaldiz, Cristina Blasco-Suñe

**Affiliations:** 1 Professor, Director of Department of Ophthalmology, University Hospital Sant Joan, University Rovira i Virgili, Institut de Investigació Sanitaria Pere Virgili (IISPV), Reus, Spain;; 2 Fellow of Department of Ophthalmology, University Hospital Sant Joan, University Rovira i Virgili, Institut de Investigació Sanitaria Pere Virgili (IISPV), Reus, Spain;; 3 Senior Ophthalmologist Department of Ophthalmology, University Hospital Sant Joan, University Rovira i Virgili, Institut de Investigació Sanitaria Pere Virgili (IISPV), Reus, Spain;; 4 Fellow of Department of Ophthalmology, University Hospital Sant Joan, University Rovira i Virgili, Institut de Investigació Sanitaria Pere Virgili (IISPV), Reus, Spain

**Keywords:** Laser, grid laser, focal laser, pan-retina-photocoagulation, anti-VEGF injections, diabetic retinopathy, diabetic macular edema, clinically significant macular edema, diffuse macular edema, focal macular edema.

## Abstract

Diabetic macular edema (DME) is the leading cause of blindness in the diabetic population. The diabetes Control and Complications Trial reported that 27% of patients affected by type 1 diabetes develop DME within 9 years of onset. Other studies have shown that in patients with type 2 diabetes, the prevalence increased from 3% to 28% within 5 years of diagnosis to twenty years after the onset. At the present time, despite the enthusiasm for evaluating several new treatments for DME, including the intravitreal therapies for DME (e.g., corticosteroids, and anti-VEGF drugs), laser photocoagulation remains the current gold standard and the only treatment with proven efficacy in a wide range of clinical trials for this condition. Despite being the standard technique for comparison and evaluation of the emerging treatments, we have generally poor understanding of the ETDRS recommendations, and we often forget about the results of laser in DME. The purpose of this review is to update our knowledge on laser photocoagulation for DME with an extensive review of the ETDRS results and discuss the laser techniques. Furthermore, we will describe the new developments in laser systems and review the current indications and results. Finally, we will discuss the results of laser treatments versus the current pharmacological therapies. We conclude by trying to provide a general overview that which laser treatment must be indicated and what types of lasers are currently recommended.

## INTRODUCTION

Diabetes is a chronic disease that typically causes changes in the small vessels of the whole body, changes that are referred to as diabetic microangiopathy. The ocular form is called diabetic retinopathy (DR). Approximately 25% of the people with diabetes have at least some form of diabetic retinopathy, and the incidence increases with the duration of the disease [1]. Eye diseases in diabetic population are the leading cause of blindness in adults under 75 years of age in developed countries [2]. There are two main complications of DR that cause visual loss: the proliferative diabetic retinopathy (PDR) and the presence of diabetic maculopathy [3]. 

Diabetic maculopathy may appear in two forms: 

1) Diabetic macular edema (DME)

2) Diabetic macular ischemia (DMI)

DME is defined as an accumulation of fluid between the outer plexiform and the inner nuclear layers, as well as a swelling of the Müller cells of the retina, causing expansion of the retinal extracellular space, in some cases involving the intracellular, both in the macular area. The prevalence of DME is higher in type 2 (DM2) than that in type 1 (DM1) diabetic patients. Our study group in 2007 found a prevalence of DME of 12.9% in DM2 patients and 7.86% in DM1 patients [4].

The Diabetes Control and Complications Trial (DCCT) [5] reported that 27% of DM1 patients develop DME within 9 years of diabetes onset. Other studies have shown that in patients with type 2 diabetes, the prevalence increased from 3% to 28% within 5 years of diagnosis to twenty years after the onset [6]. DME tends to be a chronic disease, although it is important to recognize that about 33% to 35% of patients with DME resolve the condition spontaneously after 6 months [7, 8]. The edema in the macular area occurs secondary to an abnormal permeability of the capillaries surrounding the macula (failure of inner retinal blood barrier), and in turn to a failure in the outer retinal barrier (formed by the retinal pigmented epithelium). These two mechanisms are responsible for the accumulation of interstitial fluid at the macula [9, 10]. 

While there is currently no treatment for DMI, there are different treatments for patients with macular edema, including the photocoagulation treatment with focal or grid laser, which remains the gold standard of treatment for DME. In recent years new treatment regimens with intravitreal corticosteroid or anti-VEGF injections and anti-VEGF drugs, and combined treatments of laser and intravitreal injections have been studied. Finally, in cases where vitreous traction is demonstrated, the treatment of choice is to perform a posterior vitrectomy surgery (VPP). The use of the laser source as a method of treatment for DME was first evaluated in a protocol within the Diabetic Retinopathy Study (DRS) in 1981 [11]. The effectiveness of the xenon arc source and argon laser light in the treatment of proliferative diabetic retinopathy was verified, with a reduction in visual acuity of less than 5/200 in 50% of cases, and with a stability of visual acuity for at least 4 months. The next clinical trial of diabetic retinopathy, the Early Treatment Diabetic Retinopathy Study (ETDRS) evaluated the efficacy of laser treatment in 3.711 patients, assigning patients randomly into two groups, the first receiving laser treatment immediately and the second, subjected to treatment with aspirin and laser, being delayed until five years [11-14].

The ETDRS results suggested that scatter laser photocoagulation should be considered for all eyes with severe nonproliferative diabetic retinopathy or worse, because the rate of severe loss was reduced by more than 50% of those treated with early laser photocoagulation compared with eyes assigned to deferred laser photocoagulation. Regarding macular edema treatment, the ETDRS, further concluded that focal or grid laser photocoagulation was effective [13, 15]. Despite the fact that ETDRS study has been the gold standard in the classification and treatment of diabetic retinopathy and macular edema, it seems that DME photocoagulation laser treatment has been replaced by the new intravitreal drugs. This work aims to review the knowledge we currently have on the importance of laser photocoagulation, the different techniques and laser sources, and the current indication in patients with DME.

## DEFINITION OF MACULAR EDEMA AND CLINICALLY SIGNIFICANT MACULAR EDEMA

In clinical care we use two different terms to define macular edema secondary to diabetes mellitus [16, 17]: 

1) Diabetic macular edema (DME)

2) Clinically significant macular edema (CSME) 

Both terms are different and are a source of confusion. In many studies, the terms are used indifferently and have led to confusing results. 

Diabetic macular edema (DME) is defined as retinal thickening (associated with the typical lesions such as microaneurysms, retinal edema and hard exudates) within 1 disc diameter from the foveal centre and with two disc diameters wide (1 disc diameter = 1500 μm); it can either be focal or diffuse in distribution.

Clinically significant macular edema (CSME) is a form of DME that was precisely defined by the ETDRS [32] as any of the following criteria being met:

1) Any retinal thickening within 500 μm of the centre of the macula.

2) The presence of hard exudates at or within 500 μm of the centre of the macula, if associated with thickening of the adjacent retina (not residual hard exudates remaining after the disappearance of retinal thickening)

3) A zone, or zones, of retinal thickening 1 disk area or larger, any part of which is within 1 disk diameter (1 disc = 1500 μm) of the centre of the macula.

**Fig. (1):**
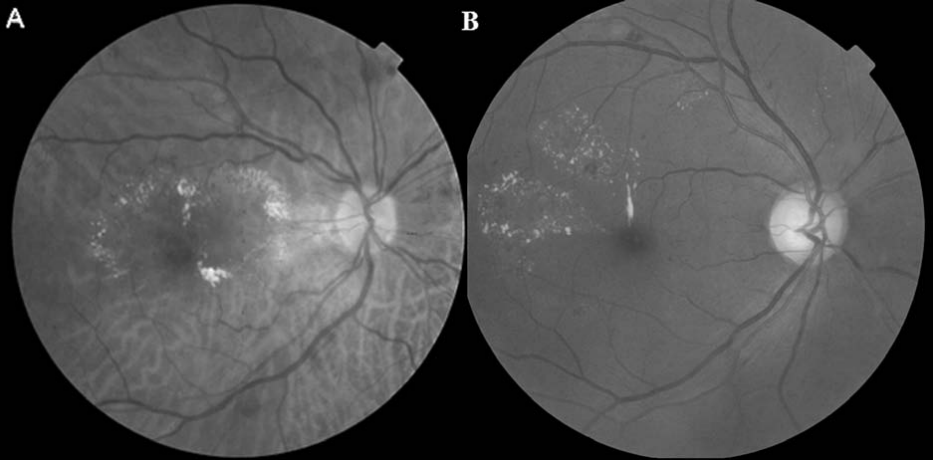
Red-free fundus photography showing diffuse diabetic macular edema.

The most useful classification used for clinical diagnosis, and subsequent DME treatment, is based on macular distribution [18, 19], which classifies them as focal or diffuse macular edema:

1) Focal macular edema is associated with circinate rings of hard exudates resulting in leakage from microaneurysms that would lead to macular edema. Focal macular edema can be unique, with only one focus of macular edema, or multi-focal (with more than one focus).

2) Diffuse macular edema represents a more extensive breakdown of the blood retinal barrier with leakage from both microaneurysms and retinal capillaries (Fig. **1**). This type is observed during late hyperfluorescence angiography of a significant size (typically more than two papillary diameters) with scarce microaneurysms and hard exudates.

## DIFFERENT LASERS USED IN OPHTHALMOLOGY

Classification of the ocular tissue lesion produced by laser [20]

1) Photocoagulation: thermal effect. Lesions are caused by an increase in tissue temperature, causing vaporization of liquids within and outside tissues and denaturalizing proteins, resulting in cellular death (apoptosis). Lasers of this kind are called photocoagulators, some of which are of argon (514.4 green and 488 blue-green nanometers) and krypton (647.1 red nanometers), which require a water refrigeration system. Currently, the most commonly used are double-diode (532 nanometers) and double-YAG (532 nanometers), which do not require cooling systems.

2) Disruption: electromechanical effect. These lasers use a burst of optical pulses of high power and short duration, achieving ionization of the tissue, forming plasma that expands at high temperature, which causes an acoustic shock wave that breaks the target tissue. Lasers of this type are called photodisruptors and Neodimio YAG (Yttrium-Aluminum-Garnet) operated with a longitudinal wave of 1064 nanometers is the one most commonly used. Its usefulness is in carrying out a capsulotomy after opacification following cataract surgery, and peripheral iridotomy to prevent risks of acute angle-closure glaucoma.

3) Photochemical, in which the lasers are used to alter the chemical composition of the target tissue, producing a molecular alteration of the cells subjected to a prior photosensitization. This type of treatment is called photodynamic therapy. In this type of laser, the treatment is carried out by photosensitization of the tissues, using photosensitizing agents like verteporfin (with a laser light absorption peak of 689 nm), which binds to the lipoproteins LDL-cholesterol. The activation is done by a non-thermal diode laser of 689 nm for 83 seconds, giving a dose of 50 jules/cm^2^ luminous light intensity of 600mW/cm^3^. Once activated, verteporfin radicals release oxygen, a process that alters the membranes of endothelial cells of the ocular blood vessels. This produces a platelet aggregation and forms a thrombus, resulting in an occlusion of the vessels. This technique is currently used in the treatment of exudative AMD (age related degeneration).

4) Photodecomposition. This is the result of the interaction of the laser with the tissues, which emits ultraviolet light at the target tissue. In this case, the laser photons are absorbed at the molecular level, resulting in fragmentation of the molecules. They emit ultraviolet radiation of 193 nanometers in pulses of 10 nanoseconds and the emitted radiation destroys molecular unions forming a volatile phenomenon called photodecomposition. The Excimer lasers (Argon - Fluoride) used in refractive surgery sculpt the corneal stroma using the photoablation.

Of the lasers described above, the treatment of DR and the DME appearing there is carried out with photocoagulator lasers. The first of their type used were argon and krypton, which were very cumbersome owing to the need for water cooling facilities. These have been replaced by the so-called solid lasers, which use diode or YAG, doubled to a produced radiation of 532 nanometers.

One type of laser also used in DME is the diode of 810 nanometers and acts in micropulses of 0.1 ms duration. 

## PHOTOCOAGULATION

The initial laser used in retina treatment with a thermal effect was the xenon arc photocoagulator. It is, in fact, not a true laser; it was introduced by Meyer-Schwickerath [21, 22] who, in a large series of publications, demonstrated in proliferative diabetic retinopathy the effectiveness of light burns over new vessels. This technique changed to a long, slow, moderately intense burning, turning the retina white adjacent to the new vessels, and sometimes causing them to narrow, slowing the flow within them. A true laser was made later; the first to be introduced was the argon laser. This produced a blue-green beam with sufficient intensity to reproduce the effects of a xenon arc with more intensity and a narrower beam. At the same time, the ruby laser was being evaluated [23]; the long wavelength and very brief exposure time of the ruby laser limited burns mainly to the outer layers of the retina, without immediate visible effects in new vessels, leading to the abandonment of the technique. 

Diabetic retinopathy and diabetic macular edema are treated by lasers that produce thermal photocoagulation. As commented, the first laser used in retina treatment was the argon laser, which was then followed by the red krypton laser and finally the dye laser (which allowed the light to change from yellow to green, according to the needs of the retina. These types of lasers have been changed for solid lasers (doubled-diode or doubled-YAG). Unlike the argon and krypton lasers, the doubled diode or YAG lasers are much more power efficient, allowing them to be connected to standard power outlets available in any hospital or clinic. The laser emission is located in the green 532 nm, being much more effective than conventional argon lasers. The tissue response at wavelength 532 nm is more similar to that with the dry yellow-green argon (514 nm) and almost the same as the Krypton yellow (568 nm). Compared with the argon (514 nm) laser, doubled diode or YAG lasers have higher absorption of oxyhaemoglobin (HbO) and haemoglobin (Hb), less dispersion (the long wavelength) and low absorption of xanthophyll pigment.

The first clinical trial was initiated by a British multicentre research that used xenon arc photocoagulation [24], and later by the National Eye Institute’s Diabetic Retinopathy Study [25], known as the DRS, which compared xenon arc and argon laser photocoagulation. The DRS studied patients with proliferative diabetic retinopathy in at least one eye or severe non-proliferative DR in both eyes, with a visual acuity of at least 20/100 in each eye. Patients were assigned to xenon arc or argon laser photocoagulation treatment, and followed at four months intervals. At two-years follow up, the DRS concluded that prompt laser treatment for eyes with severe non-proliferative DR or proliferative DR was effective. Furthermore, the DRS concluded that because the harmful effects were higher with xenon arc than argon laser, the latter laser was a preferable treatment for diabetic retinopathy [26].

### Laser Effect Mechanism

The effect of laser photocoagulation on the retina for diabetic retinopathy is still unknown, although different explanations have been put forward. The first is the occlusion of microaneurysms. In focal laser treatment in cases of focal macular edema, it is thought that direct microaneurysm photocoagulation around macular area reduces the leakage from the MA with a consequent decrease in macular edema. However, in the grid laser treatment technique, this mechanism might only function partially, so other possible mechanisms have been suggested:

1) Oxygen increases through the laser scar. One explanation involves laser-induced destruction of oxygen-consuming photoreceptors; the laser scars produce an apoptosis of photoreceptors, retinal pigment epithelium and choriocapillaries, and the scars allow oxygen (that normally diffuses from the choriocapillaries into the outer retina) to diffuse through the laser scar into the inner retina, thus relieving the inner retinal hypoxia [27].

2) A decrease in autorregulatory vasoconstriction. In diabetic retinopathy (also in DME), retinal vascular perfusion is increased with an arteriolar and venular dilatation. Following laser photocoagulation, Gottfredsdottir et al found that arteriolar branches constricted by 20.2% and the venular branches by 13.8%. The authors hypothesized that the improved retinal oxygenation leads to autoregulatory vasoconstriction with subsequent improvement in the DME [28].

3) A decrease in the whole area of abnormal leakage. Wilson et al demonstrated a reduction in the retinal capillary area in the laser photocoagulation zone, and suggested that when the area of abnormal leaking vessels is reduced, the amount of leakage would be reduced, which would result in the macular edema being resolved [29].

4) Restoration of retinal pigment epithelium (RPE) barrier. The RPE cells might respond to the laser injury in several ways: if the lesion is small (<125 µm) the RPE defect can be filled by spreading, but if the defect is relatively large the RPE cells proliferate to resurface the area, and the new RPE cells produce cytokines (*e.g.* TGF-β) that antagonize the effects of VEGF (the most important vasculogenic molecule, implicated in DME production) [30, 31].

## DIABETIC MACULAR EDEMA, TREATMENT TECHNIQUES

Laser treatment was defined by the ETDRS study in its Reports number 3 and number 4, [32-34]. According to the ETDRS, there are two different techniques: 

*Focal* *Laser*, Focal treatment is required for focal lesions located between 500 and 3000 µm from the centre of the macula. The term ‘focal lesions’ according to the ETDRS classification includes: microaneurysms, intraretinal microvascular abnormalities (IRMA) and short capillary segments that show focal fluorescein leakage. The treatment consists of burns of 50 to 100 µm of moderate intensity and 0.05 to 0.1 second duration, the end point of treatment is whitening or darkening of focal lesions. Microaneurysms below 40 µm in diameter had successful results with low laser intensity, but microaneurysms with more than 40 µm diameter needed more intense laser burns (a more whitening result) and sometimes needed a re-treatment. The clusters of microaneurysms, in particular those with hard exudate rings, may be treated with larger spots (200 to 500 µm), with subsequent re-treatment of any large microaneurysms within the cluster with 50 µm spots to obtain darkening or whitening. The treatment of lesions of more than 3000 µm from the centre is recommended if prominent leaks are present and associated with retinal thickening or hard exudates that extend closer to the center (Table **1**).

**Fig. (2):**
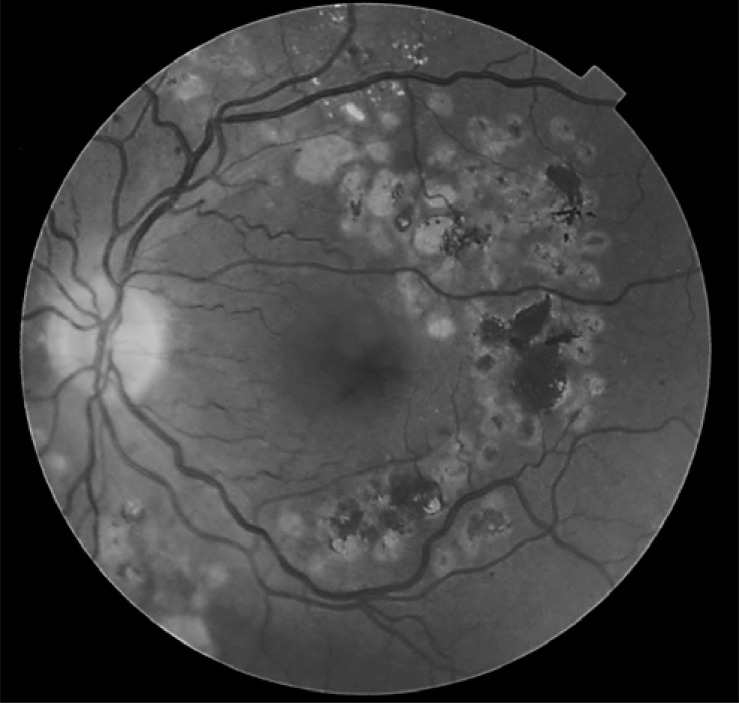
Macular fibrosis secondary to a grid laser treatment. Spectral Domain-OCT image shows the subretinal fibrosis. The red-free fundus photography shows a scar located at fovea, surrounded by the laser impact sites.

*Grid laser*, in which mild power laser impacts were made with a spot size of 50 to 200 µm, for a duration of 0.05 to 0.5 sec obtained a mild retinal pigment epithelium whitening, with power adjustment to prevent the burns from spreading to more than 200 µm in diameter. Grid treatment is not placed within 500 µm of the center of the macula or within 500 µm of the disc margin, but may be placed in the papillomacular bundle. Grid can extend up to 2 disk diameters (3000 µm) from the centre of the macula or to border panretinal photocoagulation treatment, if present (Fig. **2**). Any focal leaks within the areas of the grid treatment are treated focally. The laser burns are placed approximately two visible burn widths apart in the areas of the macular edema (retinal thickening) that are thought to be related to diffuse leakage or capillary loss (Table **1**). 

### Mild Macular Laser Photocoagulation (MMG)

This new approach to macular laser photocoagulation has recently become the focus of interest for ophthalmologists. In this new method, the burns are applied to the entire area (as described below) for treatment (including unthickened retina). Burns are focused/located over 500 to 3000 microns above, nasally and under the center of macula, and 500 to 3500 microns towards the temples [35]. There are no burns within 500 microns of the disc. The burn intensity of the grid laser is barely visible (light grey); 200 to 300 burns in total are distributed evenly over the treatment area (approx. 2 to 3 burn widths apart). The MMG burns are lighter and more diffused in nature and are distributed over the whole macula in both areas of thickened and unthickened retina. Microaneurysms are not directly photocoagulated (Table **1**). In contrast, the ETDRS focal/grid photocoagulation comprised of treating only areas of thickened retina (and areas of retinal nonperfusion) and leaking microaneurysms. The Diabetic Retinopathy Clinical Research Network (DRCR.net) compared this technique [35] with the previously described modified-ETDRS gold standard technique. Between July 2003 to October 2004, 263 patients (with a total of 323 eyes) were enrolled and assigned randomly to each technique (n = 162 eyes to the mETDRS technique and n = 161 eyes to the MMG technique). Despite the hopes for this new method, there was no indication that the eyes treated with MMG had a better outcome after 12 months of follow up than those receiving mETDRS treatment. In fact, eyes in the mETDRS group experienced a slightly greater reduction in retinal thic-kening and a trend towards a slightly better visual acuity out-come. In conclusion, despite potential advantages in theory, after 12 months of follow up the MMG laser technique is less effective in reducing OCT measure retinal thickening than the mETDRS technique frequently used in current clinical practice. Having said that, the visual acuity outcomes with either approach are not significantly different. This study does not therefore provide data to suggest that a larger long-term trial of the MMG technique is likely to show substantial clinical benefit over the current mETDRS approach.

### Subthreshold Diode Micropulse Laser Photocoagulation (MPD)

This recent technique uses a subthreshold laser micropulse, using an 810 nanometre diode laser; the desired effect is to reduce the laser damage to ocular tissue; its application in the macular area is very promising in order to treat DME with the less retinal damage. Although conventional photocoagulation is a destructive procedure, chorioretinal damage can be minimized by modifying laser parameters and clinical endpoints in the following ways: by decreasing wavelength, spot size, retinal irradiance or pulse duration. In continuous wave mode, the laser energy is delivered as a single pulse, with a typical width in the range of 0.1-0.5 seconds exposure. In micropulse mode, the laser energy is delivered with a train of repetitive short pulses (typically 100x300 msec. each) in packets. The greatest limitation of MPD laser procedures is the difficulty of the treatment without the feedback of an ophthalmoscopically visible endpoint. Conversely, minimizing chorioretinal laser damage allows confluent therapy and re-treatment of the same areas, which may be needed in macular edema. Re-treatment is feasible after MPD, because it does not produce chorioretinal scars that might expand or increase the risk of choroidal neovascularization. The treatment protocol is not yet well established in terms of the exact laser irradiance (power per unit of area) that should be delivered to the retina.

The introduction of the infra-red diode laser and its proven efficacy in treating DME has provided a valuable insight into the mechanism of action of retinal laser therapy. Direct closure of microvascular abnormalities with a relatively heavy burn is not necessary to achieve the desired clinical therapeutic endpoint. Micropulsed diode laser therapy has laid further weight to this concept, with an increasing body of clinical evidence suggesting that resolution of retinal vascular pathology is possible with low energy, subthreshold lesions. 

The literature reports some studies of its uses; the first published data was by McHugh [36], who showed a clinically significant burn in the pigment epithelium by photocoagulation with a diode laser (810 nm). However, it caused less damage to the retina than argon laser photocoagulation, and the pain associated with treatment was reported as comparable. In another study Ulbig [37] showed that CSME was completely or partially resolved in 82% of eyes treated with a diode laser, and visual acuity deteriorated in 3% of treated eyes after 6 months of follow-up. Finally, Akduman [38] compared the diode and argon green laser treatment for diffuse DME. Results at the 24-month follow-up showed that 92% of eyes treated with the diode laser and 95% of eyes treated with the argon green laser had complete or partial resolution of the macular edema, and the visual acuity remained unchanged in 75% of eyes treated with the diode laser and in 74% of eyes treated with the argon green laser. Further studies have been published reviewing the protocol of applying subthreshold micropulse diode laser photocoagulation and describe the parameters needed for DME treatment [39, 40]. There are currently not enough evidence-based clinical practice guidelines for its use, therefore further large studies are required using this technique before a recommended new treatment for DME can be established.

### Follow up After Laser Treatment

The ETDRS indicates when treatment is needed for new lesions or recurrent leakage in macular area. A new treatment is recommended when clinically significant macular edema and lesions suitable for focal or grid treatment are present. New treatment can be carried out at a six-week follow up visit if it is apparent that treatable lesions have obviously been missed during the initial treatment. Further treatment can be delayed until the next four-monthly visit, and each subsequent 4-monthly follow up visit. At all these visits, treatment can be repeated if macular edema persists and involves the centre of the macula, that means, there is a presence of clinically significant macular edema.

## LASER PHOTOCOAGULATION COMPLICATIONS

Laser photocoagulation is not a harmless technique. The laser burn induced in the retinal layers, in particular the destruction of the retinal pigment epithelium, might lead to apoptosis of the surrounding retinal cells. In the macular area, this secondary effect might affect the visual acuity.

**Fig. (3):**
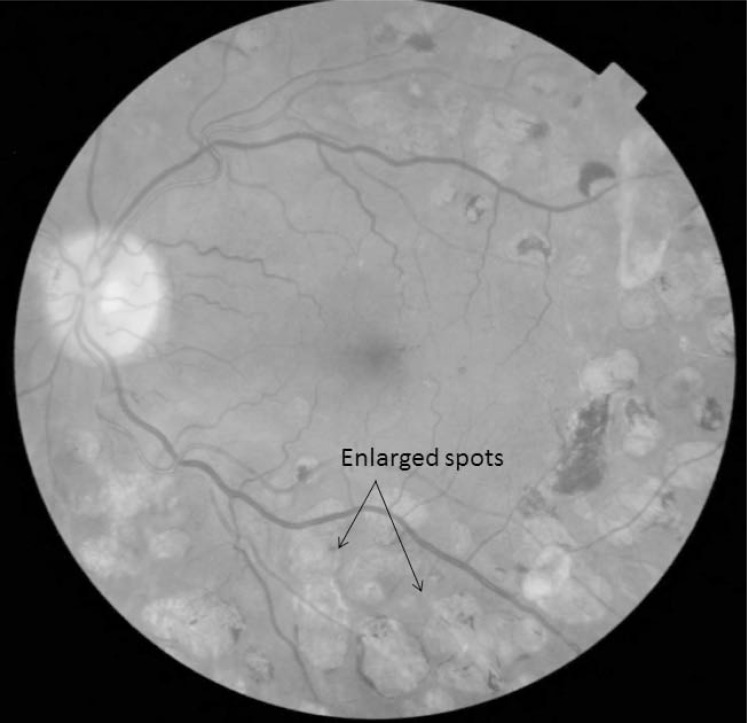
Retinal fundus photography after ten years of a laser treatment. There is an observable increase in the size of the initial laser spots, and a lot of hyperpigmented areas can be seen in the laser scars.

One of the most important effects that can reduce visual acuity is the enlargement of a laser scar, referred to as ‘atrophic creep’, as we can observe in (Fig. **3**), where the initial laser scars increase in size and coalesce each other, with a great hyperpigmentation within them. This coalescence of the laser scars might threaten the visual prognosis if the laser is applied too close to the fovea. Schatz [41] reported that enlarged laser scars reached the central fovea in 11 of 203 eyes with diabetic macular edema after grid laser photocoagulation. Brancato [42] reported that the scars enlarged by an average of 103% after treatment of choroidal neovascularization in degenerative myopia. Shah [43] observed expansion of laser scars after treatment of extrafoveal choroidal neovascularization associated with ocular histoplasmosis syndrome in 34 patients. For a subgroup of 18 patients who had a 2-year follow-up visit, the average chorioretinal scar expanded 50.1% per year for the first 2 years and 4.6% per year thereafter. The Maeshima study [44] showed that the expansion rate of laser scarring was higher in the posterior pole (12.7%) than in the midperiphery (7.0%). The authors explain that because the density of the photoreceptors is higher in the posterior pole, more photoreceptors are destroyed in the posterior pole than in the midperiphery when using the same spot size of laser photocoagulation. Furthermore, the photoreceptors interact with surrounding photoreceptors through horizontal or amacrine cells; thus, the authors hypothesized that necrosis of regional photoreceptors may lead to apoptosis of surrounding cells, which might explain why laser scars gradually expand at a higher rate in the posterior pole. The same authors indicate that the expansion rate was even higher 4 years after treatment, and that lasers with longer wavelengths contributed to larger areas of chorioretinal atrophy compared to lasers with shorter wavelengths.

**Fig. (4):**
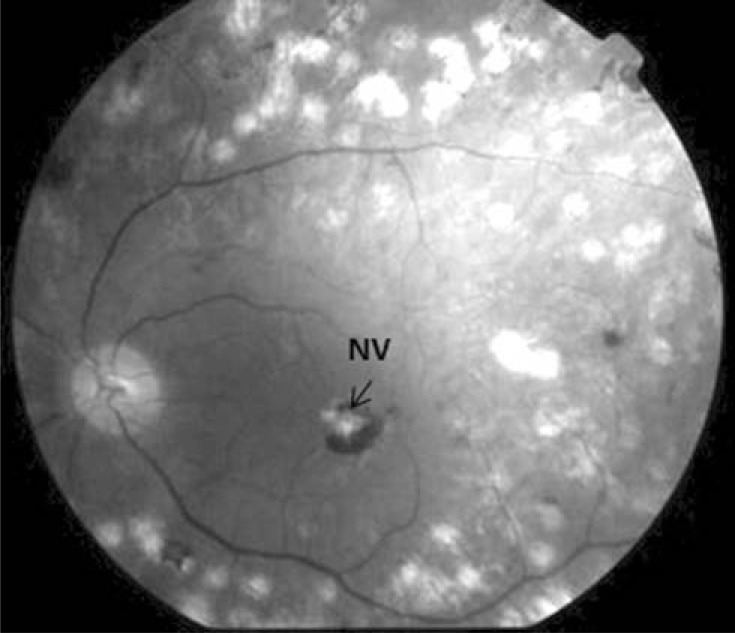
In this image we can observe an epiretinal membrane, which tractions the macula to the laser burns.

If the laser burn affects the Bruchs’ membrane, a choroidal neovascular membrane can grow under the neurosensorial retina in the burn scar (Fig. **4**). 

This serious complication that follows laser treatment for DME might be due to the use of repeated small-size, short-duration lasers, or intense laser burns, or both. These membranes can enlarge and cause a decrease in visual acuity secondary to destruction of the retina. This neovascular membrane has a good response to intravitreal antiVEGF agents [45]. Other secondary effects are those generally encountered in retina laser photocoagulation, such as photophobia, and an appearance of scotomas in the visual field.

## CLINICAL TRIALS OF DIABETIC MACULAR EDEMA LASER PHOTOCOAGULATION 

Macular edema laser photocoagulation is currently the gold standard for all comparative new drug treatment studies. All new intravitreal anti-VEGF drugs have been compared to focal or grid laser treatment. The first extended clinical trial was The Diabetic Retinopathy Study Research Group (DRS), although the gold standard study is the ETDRS.

### The Diabetic Retinopathy Study (DRS)

This was the first longitudinal study on the efficacy of laser photocoagulation in DR patients. In this study, DR was first classified according to the Airlie House (later modified by the ETDRS), which allowed us to study the changes in diabetic retinopathy status after a lapse of time or after treatment [46]. The study enrolled 1742 patients with proliferative DR in at least one eye or severe non-proliferative DR in both eyes, with a visual acuity of at least 20/100 in each eye [47]. Each patient was randomly assigned to either the argon laser or xenon arc treatment group; one eye was randomly assigned to photocoagulation treatment and the other to indefinite deferral treatment. The argon laser technique specified 800 to 1600, 500 µm scatter burns of 0.1 second duration and direct treatment of new vessels on the disc and elsewhere, whether flat or elevated. The xenon arc treatment was similar, but burns were fewer, of longer duration, and stronger, and direct treatment was not applied to elevated new vessels or those on the surface of the disc [48]. As its principal variable, the DRS termed visual acuity less than 5/200 as ‘severe visual loss’. This visual acuity level was chosen as the level at which vision becomes too poor to be useful for walking about or for other self-care activities [47].

The results at four years did not differ from those at 2 years follow up and the DRS concluded that the treatment by argon laser or xenon arc was more effective than no treatment at all or deferral treatment [49, 50]. Regarding the preservation of eyes from visual loss, the xenon arc group was more effective than the argon laser group, but harmful effects (defined as a decrease of one or more lines of visual acuity, and constriction of peripheral visual field) were higher in the xenon arc group, concluding that argon laser photocoagulation should be the first line treatment technique for eyes with proliferative DR or severe DR [26].

Furthermore, the DRS identified four retinopathy risk factors that increase the 2-year risk of developing severe visual loss [50]: 1. The presence of new vessels, 2. new vessels located on or within 1 disc diameter of the disc (NVD), 3. new vessels moderate to severe (NVD equaling or exceeding those in standard photograph 10A or for eyes without NVD, NVE equaling or exceeding one half disc area in at least one photographic field), and 4. vitreous or preretinal hemorrhage. These four risk factors defined the eyes with high-risk characteristics in the proliferative DR group of patients, a definition that is still currently used.

Despite the DRS having focused its results on the proliferative DR treatment, the presence of DME was also studied, because in patients treated by argon laser or xenon arc, DME appears or if it is present at baseline the DME worsens. The DRS is the first study to recommend combining focal macular laser with the scatter laser treatment, but in the first episode of photocoagulation the scatter laser should be combined only at the nasal quadrants, and treatment of the temporal quadrants should be delayed for future photocoagulation sessions [26, 51]. 

### Early Treatment Diabetic Retinopathy Study (ETDRS)

The Early Treatment Diabetic Retinopathy Study research group (ETDRS) was a longitudinal study that enrolled 3711 patients from April 1980 to July 1985. Patients were allocated to two groups: Group 1 patients had no macular edema, visual acuity of 20/200 or better, and mild, moderate or severe non proliferative DR, or early proliferative DR. Group 2, which is of interest to this study, comprised of patients with DME, visual acuity of 20/200 or better, and mild, moderate or severe non proliferative DR [12]. In the patients with DME, the investigators were randomly assigned one eye of each patient to early photocoagulation (focal or grid), and the other eye was assigned to deferral of photocoagulation; the follow up visits were scheduled at four months intervals [15].

The patients with DME were sub-classified in three groups: 1) patients with DME (not clinically significant), 2) patients with clinically significant macular edema (CSME) with retinal thickening without centre of the retina involvement, and 3) patients with CSME and retinal thickening of the centre [52].

Subjects were given laser photocoagulation immediately (713 eyes) or deferred (1409 eyes). The investigators evaluated the visual loss at 12, 24 and 36 months in the follow up.

Visual loss was defined as a loss between baseline and follow up visit of 15 or more letters in the ETDRS optotypes, equivalent to a doubling of the initial visual angle (*i.e.* change of 20/20 to 20/40, or change of 20/50 to 20/100). In the results of the ETDRS at 36 months, visual loss was reported in about 65% of eyes that were not treated, in 33% of eyes whose treatment was deferred and in only 13% of the eyes submitted for immediate laser treatment. The study concluded that immediate laser treatment is effective in eyes with DME [14, 15, 52]. From those results, DME laser photocoagulation became the gold standard, and since then all new treatments have been compared with it.

One important finding of the ETDRS was that the effect of DME laser photocoagulation increases over time thus in eyes with CSME, visual acuity increases by about 1% in the first year, 6% at 24 months and 10% at 36 months.

### Laser Results in the New Drugs Studies

The introduction of intravitreal anti-VEGF and corticoids (triamcinolone) in DME treatment changed the current treatment protocols. Studies have compared the effectiveness of new drugs with that of the laser (focal/grid) effect; in all studies a control group submitted to laser photocoagulation has been the gold standard. The following section presents the results of the four most important studies. 

### Clinical Results from Other Published Studies 

Many other studies have shown the beneficial effect of photocoagulation therapy for DME (Table **2**). All of these studies were clinical series, and the results were presented at two years follow up [53-60] and showed similar results to the ETDRS. It is interesting that Karacolu [59], who carried out a study at one-year follow-up, reports no improvement in visual acuity (VA) in his series against other studies that report a percentage between 8.3% to 25% of improvement after two or three years follow up. The relative weakness, in these series is the small number of eyes included, apart from the Lee study [58].

Ranibizumab Monotherapy or Combined with Laser Versus Laser Monotherapy for Diabetic Macular Edema (the RESTORE study).

This phase III study compared three groups of eyes: Group 1) those given only a ranibizumab injection, Group 2) those given ranibizumab and the laser, and Group 3) a control group of eyes treated only by laser [61]. Regarding the VA gain at 24 months, 8.2% of eyes in Group 3 achieved > 15 letters, 22.6% in Group 1 and 22.9% in Group 2. The decrease in central retinal thickness was more important in eyes in group 2 with a mean decrease of 116.5 µm, followed by Group 1 with a mean decrease of 103 µm, and Group 3 with a 60 µm decrease.

The RESTORE study made two different subgroups of patients, attending the type of diabetic macular edema (focal versus diffuse).The first subgroup was patients with focal macular edema which included 183 patients. In this subgroup of patients the mean letters score change at 12 months was an increase of 0 letters for the group submitted to laser (52 patients), in front an increase of 6 letters in the other two groups (63 patients submitted to ranibizumab injection alone, and 68 patients submitted to ranibizumab plus laser). The second subgroup with diffuse diabetic macular edema included 143 patients. In this group 52 patients were submitted to laser treatment, 45 patients submitted to ranibizumab injection alone, and 46 patients submitted to ranibizumab plus laser, as in the previous first subgroup with focal edema, the mean change of visual acuity at 12 months was 0 letters for patients submitted to grid laser alone, an increase of 6.5 letters in patients with ranibizumab injection alone, and an increase of 6 letters in the group submitted to ranibizumab plus laser.

### Bevacizumab or Laser Therapy (the BOLT Study)

This study divided the eyes into two groups: Group 1) given only a bevacizumab injection and Group 2) only laser (focal/grid) at 12 months [62]. The results show a significant difference between the mean visual acuity at 12 months in the bevacizumab group (61.3±10.4; range 34-79) and laser arm (50.0±16.6; range 8-76). Furthermore, the bevacizumab group gained a median of 8 letters, whereas the laser group lost a median of 0.5 letters. The proportion of patients who gained >15 letters was 11.9% in the bevacizumab group and 5.3% in the laser arm, with approximately one third of patients (31.0%) gaining >10 letters in the bevacizumab group compared with 7.9% in the laser arm. 

Attending the changes of the CMT, in the bevacizumab group, had significantly decreased from 507±145 µm at baseline to 378±134 µm at 12 months, whereas over the same time period in the laser arm it had decreased to a lesser extent, from 481±121 µm to 413±135 µm. 

### Ranibizumab for Edema of the Macula in Diabetes (the READ2 Study)

This study at 24 months is the most interesting for the present review of the laser effect. Patients were divided into three groups: Group 1) given only a ranibizumab injection, Group 2) those given ranibizumab and the laser (focal/grid), and Group 3) only laser (focal/grid). The increase in VA by >15 letters at six months was 7.9% in Group 1, 0.1% in Group 2 and 3.6% in Group 3, but at 24 months Groups 2 & 3 had increased by a higher percentage than Group 1 had, thus at 24 months the increase of VA was 0.3% for group 1, 4.5% for group 2 and 2.9% for group 3. Furthermore, at 24 months the eyes which had improved by more than 5 letters were: 47% for Group 3 against 36% for Group 1. Finally, the increase in the number of letters between 6 months and 24 months was: 4.6 letters for Group 3 and 3.0 letters for Group 1. We can conclude that the laser effect increases over time, and the intravitreal injection of ranibizumab has a prompt effect but with no important increase over time [63].

### Diabetic Retinopathy Clinical Research Network (the DRCR-Net Study)

This was a long study with a three-year follow-up [64-66]. Patients were divided into four groups: Group 1) submitted only for laser, Group 2) given a ranibizumab injection and prompt laser treatment, Group 3) given a ranibizumab injection but with deferred laser treatment, and Group 4) given a triamcinolone injection and laser. 

The first-year results report an increase in VA by >15 letters in 15% of Group 1, in 30% of Group 2, being the most effective, in 28% of Group 3 and in 21% of Group 4. The decrease in central retina thickness in the first year was: 79 nm, 112 µm, 111 µm, and 90 µm, respectively.

The two-year follow up reported the same increase in VA in 18% of Group 1, in 29% of Group 2, in 28% of Group 3 and in 22% of Group 4. The decrease in central retina thickness at year two was: 113 µm, 113 nm, 129 µm, and 96 µm, respectively.

The three-year follow up only reports the patients treated by intravitreal triamcinolone or by laser photocoagulation and there were three study groups: Group 1) those submitted only to focal/grid photocoagulation, Group 2) those given only an intravitreal injection of 1 mg triamcinolone and Group 3) those given only an intravitreal injection of 4 mg of triamcinolone). The reported increase in VA at 3 years slightly favoured Group 1, with the differences between groups at 3 years being of similar magnitude to the differences at 2 years. The mean change in VA from baseline to 3 years was +5 in Group 1 and 0 in the two triamcinolone groups. VA outcomes slightly favoured the laser group. Among those that completed the 3-year study, 51 (44%) in Group 1, 23 (25%) in Group 2 and 37 (38%) in Group 3 had improvements in VA of 10 or more letters from baseline to 3 years, and 14 (12%), 24 (26%), and 22 (22%), respectively, had a worsening of 10 or more letters.

Similar to the VA results, more eyes in all three groups had a reduction in OCT central subfield thickness from year two to year three than an increase. At three years, central subfield thickness was<250 microns in 75 (67%) eyes in the Group 1, 37 (43%) in group 2, and 45 (51%) in Group 3.

In the first two years follow up study, authors concluded that prompt laser treatment plus ranibizumab was the most effective treatment technique for DME. However, we have to bear in mind that the analysis of the results also concluded that laser treatment ameliorated VA at two years compared to the first year. In the third year follow up study, the authors concluded higher effect of laser compared to the intravitreal triamcinolone injections.

### Da Vincy Stduy

The DA VINCI study [67], was designed as a 52-weeks, multicentre, randomized, double-masked, active-controlled phase 2 clinical study, performed to assess safety and efficacy of Vascular endothelial growth factor Trap-Eye (VEGF Trap-Eye) in comparison with laser photocoagulation. VEGF Trap-Eye is a panisoform VEGF-A inhibitor whose binding affinity to VEGF is substantially greater than that of either bevacizumab or ranibizumab. The patients were randomly assigned in equally 1 of 5 treatment regimens in 1 eye only: 0.5 mg VEGF Trap Eye every 4 weeks; 2 mg VEGF Trap-Eye every 4 weeks; 2 mg VEGF Trap-Eye for 3 initial monthly doses and then every 8 weeks; 2 mg VEGF Trap-Eye for 3 initial monthly doses and then as-needed; or macular laser treatment by the modified ETDRS protocol. At week 24, up to 34% of VEGF Trap-Eye-treated patients gained ≥ 5 letters from baseline, up to 64% gained ≥ 0 letters from baseline, and up to 93% of patients gained ≥ 10 letters from baseline, compared with only 21%, 32%, and 68% in the laser group, respectively. Conversely, 9.1% of patients in the laser group and 4.5% of patients treated with 0.5 mg VEGF Trap-Eye lost ≥ 5 letters in week 24, whereas no patients in any of the 2 mg VEGF Trap Eye groups experienced such vision loss at this time point. Baseline values of mean CRT by group are given in. Reductions in CRT in each group were consistent with the observed improvements in visual acuity. Patients in the four Trap-Eye groups experienced mean reductions in CRT ranging from 127.3 to 194.5 µm by week 24 compared with only 67.9 µm n the laser photocoagulation group.

## DISCUSSION

The DME is a complex disease of multifactorial origin, the pathogenesis includes the existence of chronic hyperglycemia, along with the accumulation of free radicals, AGE proteins, and protein kinase C (PKC) formation, and the subsequent activation of vascular endothelial growth factors (especially VEGF-A) as well as an increase in vascular permeability. To treat DME, it is important to use the classification by Bresnick into focal or diffuse DME [9]. The first line treatment is the hyperglicemia control, accompanied by monitoring of blood pressure and lipid levels [68, 69], which permits the disappearance of macular edema in about 33% to 35% of patients [7, 8], in the other 70% of cases a treatment in the remaining 70% of cases we must establish a treatment based on laser photocoagulation or intravitreal injections of anti-VEGF.

As we have seen over the issue of exposure, treatment by laser photocoagulation of the DME currently remains as the gold standard against which the treatments that have to be developed later are compared. While the introduction of treatment with anti-VEGF intravitreal injections or steroids has been showing some superiority over the treatment carried out only by laser photocoagulation, this indication remains the principle in certain circumstances, and we must not forget the risks relating to the intravitreal injection drugs, such as endophthalmitis or retinal detachment, which, though mentioned in a relatively small percentage of publications, are serious complications following any treatments.

If we evaluate treatments with anti-VEGF, we see that they are superior in the resolution of DME, and VA improved in most studies within the first year of treatment (Table **3**). We can also observe that laser treatment has a greater effect at two years follow up, something that had already been demonstrated in clinical series conducted previously, in which treatment was assessed by laser, and the number of patients whose VA improved increased between years two and three. The evaluation of intravitreal corticoids made by the DRCR-Net study at three years compared results between two different doses of intravitreal triamcinolone against the focal/grid laser alone, and concluded that the laser treatment was more effective in the long term.

As a rule, any alternative to laser treatment should be evaluated after two or three years’ follow up and not only during the first year. In fact, in the ETDRS study the best results were achieved at three years’ follow up, indicating that all studies should plan to evaluate its effect over DME at the three-year follow up visit. There are few complications in laser treatment when it is carried out correctly, but it is important to take into consideration the increase in retinal scar size over time, and its impact on the macular area, that might cause a loss in VA over the years. Lasers on the market should indicate that the solid types, which emit at frequencies near 532 nm, are best, although the new laser diode (810 nm) may have a place in the future. Currently, there is insufficient scientific evidence on its use in the case of eyes with DME and further studies with the same protocols are important to determine which treatment should be carried out with this type of laser. 

In the currently anti-VEGF therapies, we must to have in mind that intravitreal injections have complications, as retinal detachment or endophthalmitis, despite the prevalence of these complications is very low. Moshfeghi [70] reported an incidence of 0.02%; (95% CI: 0.0114%-0.0348%) after six year experience in intravitreal anti-VEGF therapy with 60.322 injections, associated with a decrease in visual outcomes. The current indication for treatment by laser photocoagulation DME is uncertain, because out of doubt the treatment of macular edema that is located at more than 500 µm from the centre of the retina (defined as focal DME not involving the centre of the retina or impact on the area adjacent to 500 µm from the centre of the retina) is currently the best option for treatment of DME. However, the treatment of DME that involves the centre of retina has even more uncertain results.

Regarding the best drug for diabetic macular edema treatment, we currently use ranibizumab, which has been supported by many studies, bevacizumab has been widely used for DME treatment, although there is no data on the use of VEGF Trap-Eye in diabetic patients. Despite recent reports of an increase in atrophy in the macular area with the monthly use of ranibizumab [71], there have been no differences in results of the DME treatment.

Regarding the techniques used, focal treatment is widely shown to be useful in patients with focal macular edema, especially if no clinically significant macular edema is present (patients with macular edema insight the 3,000 μm from the centre of the macula, without affecting within 500 μm of the centre of the macula). Despite one of the revised studies, the RESTORE included a subgroup of patients with focal macular edema only treated with laser, and no increase of visual acuity has been observed at 12 months follow up, but because the objective of the RESTORE was not to study the effectiveness of ranibizumab or laser treatment in focal macular edema, and we can question the results in this subgroup of patients, the authors did not analyse the baseline and final characteristics of the patients in this subgroup, *e.g.* if the macular edema affected the 500 μm, if there are one or more exudative focus, or if circinate exudates affected the fovea, all these characteristics can alter the results of final vision. We think that for DME treatment, we must personalize it for each person and DME characteristics, which are the best option of treatment.

## CONCLUSION

- Laser photocoagulation remains the gold standard treatment. Its effect is most important after two years’ follow up.
- The most important current indication of laser photocoagulation is the focal diabetic macular edema.
- The grid laser photocoagulation technique may be indicated in cases of resistance or contraindication of anti-VEGF drugs.
- The association between laser photocoagulation and intravitreal anti-VEGF drugs, despite seeming to have an inferior effect to anti-VEGF alone, should be studied more extensively, in studies with three or more years’ follow up.
- New laser developments, such as the sub-threshold diode micropulse laser photocoagulation, despite seeming to be promising, needs more extensive studies.

## Figures and Tables

**Table 1.** Laser photocoagulation techniques for DME, attending the original ETDRS, modified ETDRS and the MMP technique.

	Direct/ Grid Photocoagulation Original EthRS	Direct/ Grid Photocoagulation Modified EthRS	Mild Macular Grid Photocoagulation Technique
Characteristic of direct treatment	Directly treat all leaking MA in areas of retinal thickening between 500 and 3000 microns from the centre of the macula (but not within 500 microns of disc)	Directly treat all leaking MA in areas of retinal thickening between 500 and 3000 microns from the centre of the macula (but not within 500 microns of disc)	Lighter and more diffuse in nature and are distributed throughout the macula in both areas of thickened and unthickened retina
Change in microaneurysms colour with direct treatment	Required at least a mild white burn should be evident beneath all MA	Not required, but at least a mild gray-white burn should be evident beneath all MA	Microaneurysms are not directly photocoagulated
Burn size for direct treatment	50 to 100 microns	50 microns	Not applicable
Burn duration	0.05 to 0.1 sec	0.05 to 0.1 sec	Not applicable
Grid treatment	Applied to all areas with diffuse leakage or nonperfusion within area described below for treatment	Applied to all areas with diffuse leakage or nonperfusion within area described below for treatment	Applied to entire area described below for treatment (including unthickened retina)
Area considered for grid treatment	500 to 300 microns superiorly, nasally and inferiorly from the centre of macula. 500 to 3500 microns temporally from macula centre. No burns are placed within 500 microns of disc	500 to 300 microns superiorly, nasally and inferiorlyfrom the centre of macula. 500 to 3500 microns temporally from macula centre. No burns are placed within 500 microns of disc	500 to 300 microns superiorly, nasally and inferiorly from the centre of macula. 500 to 3500 microns temporally from macula centre. No burns are placed within 500 microns of disc
Burns size for grid treatment	50 to 100 microns	50 microns	50 microns

**Table 2.** Visual acuity outcomes of laser photocoagulation treatment for DME, on studies published to date.

Author	Number of Eyes	VA Improved in Treated Eyes (%)	VA Unchanged in Treated Eyes (%)	VA Worse in Treated Eyes (%)	Follow up
Marcus [53]	33	17%	57.6%	24.2%	2 years
Fernandez-Vigo [54]	39	17%	60%	23%	2 years
Gaudric [55] DME with exsudates in macular area	16	18%	55%	20%	3 years
Gaudric [56] DME without exsudates	20	25%	78%	9.5%	3 years
Lee [57]	302	14.5%	60.9%	24.6%	3 years
Lee [58] combined to panretinal photocoagulation	52	4%	72%	24%	2 years
Karacolu [59]	85		85.1%	14.9%	1 year
Ladas [60]	42	8.3%	54.2%	37.5%	3 years

**Table 3.** Changes in the visual acuity (percentage) of patients in different studies. Values shown represent the percentage of patients who have gained at least fifteen letters.

Study	6 Months Results	12 Months Results	24 Months Results
RESTORE		9% laser 22.6% RBZ 22.9 % RBZ+ laser	
READ-2	0% laser 21% RBZ 6% RBZ+laser		18% laser 24% RBZ 26% RBZ+laser
DRCRnet		15% laser 30% RBZ+prompt laser 28% RBZ+deferred laser 21% TA+laser	
BOLT		7.9% laser 11.9% BVZ	
Da Vinci	21% laser 34% EGF-Trap 0.5 mg/q4 32% EGF-Trap 2 mg/q4 17% EGF-Trap 2 mg/q8		

RBZ = ranibizumab, BVZ = bevacizumab, TA = triamcinolone, EGF-Trap = Endothelial Growth Factor Trap-Eye, q4 = quarter for daily, q8 = eighthly for daily.

## ABBREVIATIONS

BOLT: = Bevacizumab or Laser Therapy in the management of DME
CSME: = Clinically significant macular edema
DCCT: = Diabetes control and complications trial
DME: = Diabetic macular edema
DRCR.net: = Diabetic Retinopathy Clinical Research Network
ETDRS: = Early Treatment Diabetic Retinopathy Study Research Group
READ-2: = Ranibizumab for edema of the mAcula in Diabetes study
RESTORE: = Ranibizumab monotherapy or combined with laser versus laser monotherapy for diabetic macular edema
UKPDS: = United Kingdom prospective diabetes study
VA: = Visual acuity
WHO: = The World Health Organization
